# Rewiring osteoimmune homeostasis in osteoporosis: T-cell subsets as key regulators and emerging therapeutic targets

**DOI:** 10.3389/fimmu.2026.1926935

**Published:** 2026-08-11

**Authors:** Yuan Gong, Wenxing Zeng, Yitao Liao, Xuanyu Xie, Zhichao Qin, Chao Li, Xian Zhang

**Affiliations:** 1 Graduate School, Nanjing University of Chinese Medicine, Nanjing, China; 2 Department of Spine, Wuxi Affiliated Hospital of Nanjing University of Chinese Medicine, Wuxi, China

**Keywords:** IL-17, immunotherapy, osteoimmunology, osteoporosis, RANKL/OPG, T-cell subsets, Th17/Tregs balance, Tregs

## Abstract

Osteoporosis (OP) is a chronic metabolic bone disease characterized by reduced bone mass, microarchitectural deterioration, and an increased risk of fragility fractures. Although conventional views have emphasized the imbalance between osteoblasts and osteoclasts, advances in osteoimmunology have shown that T-cell subsets regulate bone remodeling through multilayered mechanisms, including the RANKL/RANK/OPG axis, inflammatory cytokine networks, costimulatory molecules, immune checkpoints, the gut microbiota, and cellular metabolic reprogramming. Th17 cells and their signature cytokine IL-17 promote RANKL expression and amplify the NF-κB/MAPK/NFATc1 pathway of osteoclast differentiation, thereby constituting a key pathogenic component in inflammatory bone loss and postmenopausal osteoporosis. In contrast, regulatory T cells (Tregs) inhibit osteoclast formation through Foxp3-, IL-10-, TGF-β-, CTLA-4- and IDO-related pathways, thereby limiting proinflammatory T-cell activation and maintaining bone marrow immune homeostasis. Th1/Th2 cells, CD8+ T cells, γδ T cells, T follicular helper cells, NKT cells, and mucosa-associated unconventional T cells may also influence bone resorption and formation in different pathological contexts through factors such as IFN-γ, TNF-α, IL-4, IL-13, IL-21, IL-22, and membrane-bound RANKL. Drawing on the literature concerning T-cell subsets, the Th17/Tregs and Th1/Th2 balances, γδ T cells, and the RANKL/OPG axis, this review provides a narrative synthesis of current evidence about the molecular mechanisms by which T-cell subsets regulate osteoporosis-related bone remodeling, the features of immune imbalance in different types of osteoporosis, T-cell-targeted intervention strategies, and translational challenges. The aim is to provide a theoretical basis for developing precision antiosteoporotic therapies from an osteoimmune perspective.

## Graphical Abstract

**Figure:**
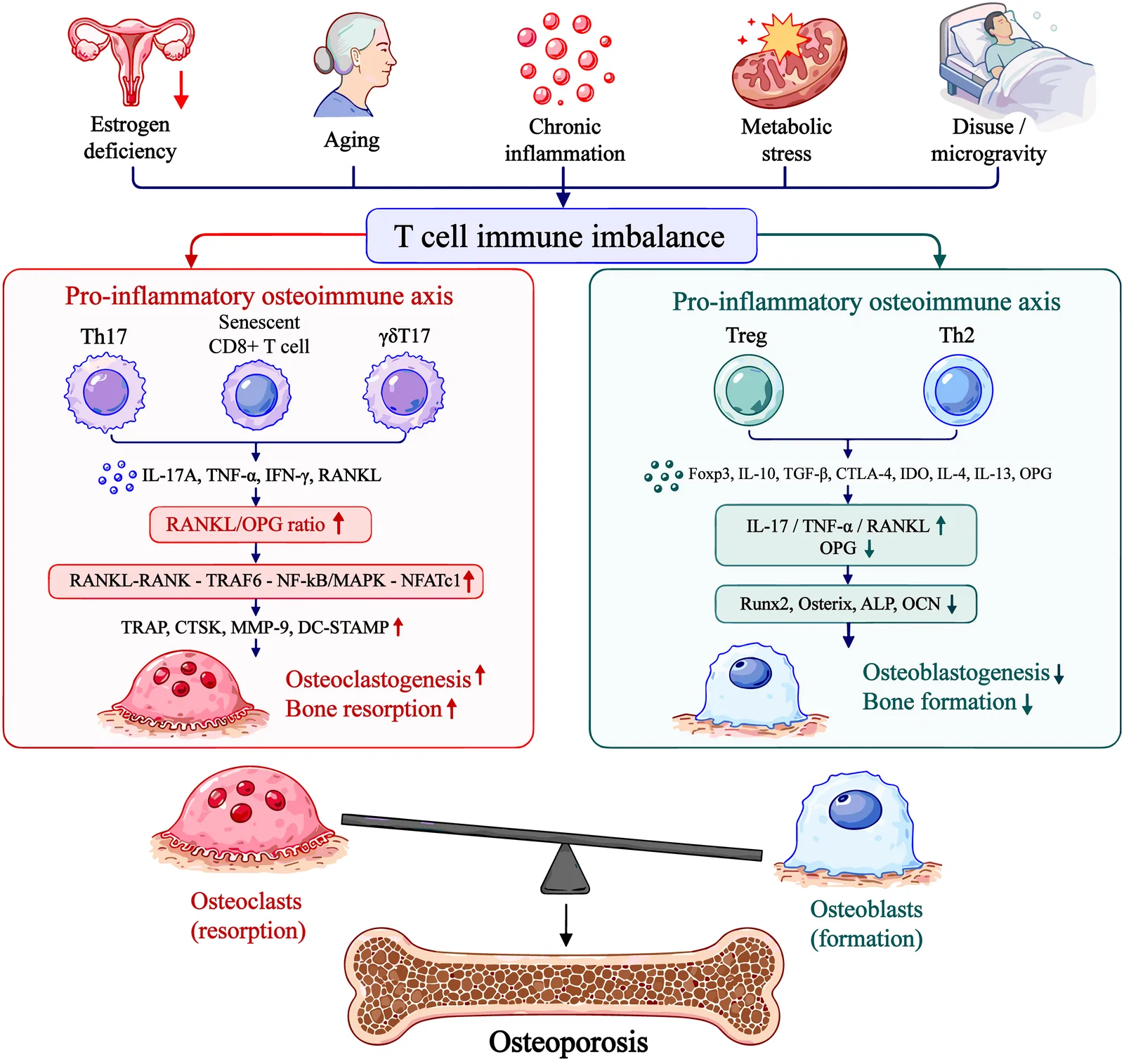

## 1 Introduction

Osteoporosis is one of the most common chronic metabolic bone diseases in the context of a global aging population, with its core pathological features including reduced bone mass, microarchitectural deterioration, reduced bone strength and increased susceptibility to fractures (1, 2). Epidemiologically, a systematic review and meta-analysis estimated that the global prevalence of OP was approximately 18.3%, with a markedly higher prevalence in women than in men—23.1% versus 11.7%, respectively (3). Analyses of the Global Burden of Disease database further demonstrated that low bone mineral density and its related fractures continue to impose a substantial health burden across 204 countries and territories (4). Among postmenopausal women, low bone mineral density was responsible for approximately 219,552 deaths and 7.76 million disability-adjusted life years worldwide in 2021, while the absolute number of attributable deaths had more than doubled since 1990, largely because of population aging (5).The vertebral bodies, hip and distal radius are common sites of osteoporosis-related fragility fractures; hip fractures, in particular, often lead to prolonged bed rest, disability, infection, thromboembolism and increased mortality, placing a substantial burden on quality of life and healthcare resources (3, 4). Current clinical management of OP is centered on antiresorptive agents, anabolic agents and dual-action drugs, including bisphosphonates, denosumab, selective estrogen receptor modulators, calcitonin, teriparatide, abaloparatide and romosozumab (6, 7). Although these drugs can significantly reduce the risk of fractures, long-term use is still associated with issues such as rebound effects upon discontinuation, medication adherence, treatment cost and rare but serious adverse reactions. Therefore, identifying more precise and sustainable immunomodulatory targets at the level of etiologic mechanisms represents a key direction in the prevention and treatment of OP.

OP is not merely a bone-cell disorder but a systemic disorder involving the interplay of endocrine, metabolic, immune-inflammatory, gut microbiota-related, and bone marrow microenvironmental factors. Estrogen deficiency, aging, impaired glucose metabolism, glucocorticoid exposure, prolonged immobilization and chronic inflammation can all alter the local cytokine milieu in the bone marrow, leading to excessive osteoclast formation and insufficient osteoblast function (8, 9). During bone remodeling, osteoclasts are responsible for the resorption of old or damaged bone, whereas osteoblasts are responsible for the formation of new bone; osteocytes, meanwhile, sense mechanical stress and regulate the coupling between osteogenesis and osteoclastogenesis via sclerostin, RANKL and other factors (10, 11). When immune cells remain in a state of low-grade activation over an extended period, bone remodeling no longer follows the physiological rhythm of bone repair but shifts toward a pathological state characterized by inflammation-driven high bone turnover or reduced bone formation.

The emergence of osteoimmunology has transformed our understanding of the pathogenesis of OP. RANKL was initially identified as a key molecular link between the immune and skeletal systems; it is expressed not only by osteoblasts, bone marrow stromal cells and osteocytes, but also by activated T and B cells (12, 13). After binding to RANK on the surface of osteoclast precursors, RANKL recruits TRAF6 and activates signaling pathways such as NF-κB, JNK, ERK, p38 and NFATc1, ultimately inducing the expression of osteoclast-related genes such as TRAP, CTSK, MMP-9 and DC-STAMP (14, 15). This pathway helps explain why chronic inflammation, immune activation and autoimmune diseases are often accompanied by bone loss, and also suggests that T cells may be a key link between inflammatory responses and enhanced bone resorption.

T-cell subsets exhibit substantial differentiation plasticity. Naive CD4+ T cells can differentiate into functional subsets such as Th1, Th2, Th17, Tregs, Tfh, Th9 and Th22 under different cytokine environments; CD8+ T cells, γδ T cells, NKT cells and other unconventional T cells can also regulate bone metabolism via cytokines, cytotoxic molecules, membrane-bound RANKL and tissue-resident properties (16, 17). Previous studies have shown that in postmenopausal patients with osteoporosis (OP) or in ovariectomized (OVX) animal models, alterations such as Th17 cell expansion, elevated IL-17 levels, reduced Tregs proportion or function, and increased pro-inflammatory factors such as TNF-α and IFN-γ are frequently observed (18, 19). Therefore, OP may be regarded as a consequence of disrupted osteoimmune homeostasis, and regulating the balance of T-cell subsets has potential therapeutic value in osteoporosis treatment.

**Figure 1:**
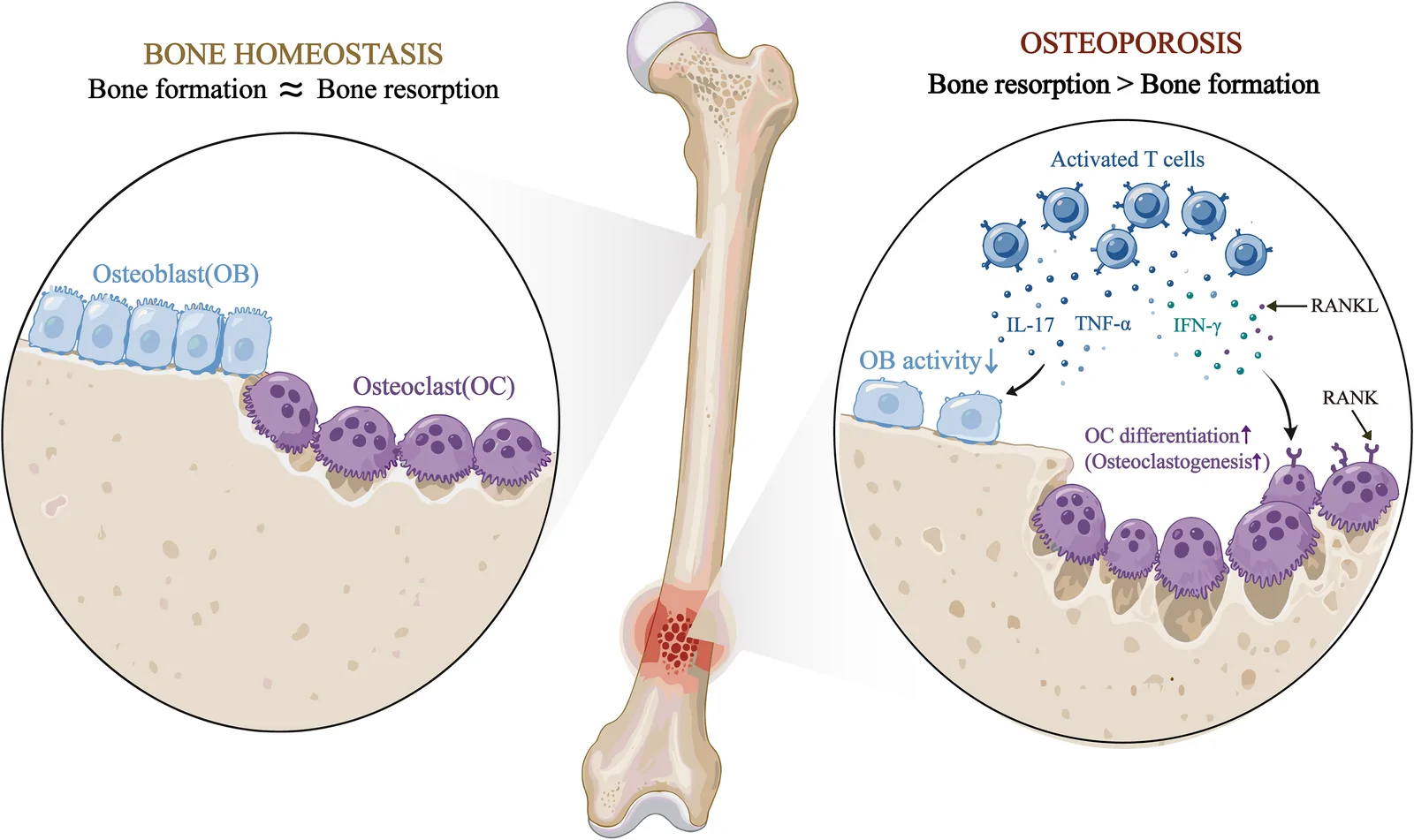
T-cell activation disrupts bone remodeling homeostasis and promotes osteoporotic bone loss. Under physiological conditions, bone formation mediated by osteoblasts is dynamically coupled with bone resorption mediated by osteoclasts, thereby maintaining bone homeostasis. In osteoporosis, activated T cells accumulate within the bone immune microenvironment and release pro-inflammatory cytokines, including IL-17, TNF-α and IFN-γ. These cytokines, together with T-cell-derived or osteoblast/stromal-cell-induced RANKL, enhance RANKL–RANK signaling in osteoclast precursors and promote osteoclast differentiation and activation. Meanwhile, inflammatory signaling suppresses osteoblast activity, shifting bone remodeling toward excessive bone resorption over bone formation. This imbalance ultimately leads to trabecular bone loss, microarchitectural deterioration and osteoporosis. OB, osteoblast; OC, osteoclast; IL-17, interleukin-17; TNF-α, tumor necrosis factor-alpha; IFN-γ, interferon-gamma; RANK, receptor activator of nuclear factor-κB; RANKL, receptor activator of nuclear factor-κB ligand.

Unlike conventional drugs that act directly on osteoclasts or osteoblasts, T-cell-targeted strategies emphasize the remodeling of the bone marrow immune microenvironment, thereby attenuating pro-resorptive signals, enhancing anti-inflammatory and reparative signals, and restoring osteoclast-osteoblast coupling. Notably, the role of T cells in bone metabolism is spatiotemporally dependent; that is, moderate inflammation in the early stages of acute injury helps clear necrotic tissue and initiates repair, whereas chronic, persistent activation leads to bone loss; factors such as IFN-γ, IL-17 and TGF-β may exhibit opposite effects depending on their dose, source and the stage of the disease (20, 21). Accordingly, this narrative review examines the mechanisms by which T-cell subsets contribute to OP and discusses the challenges associated with their precise therapeutic regulation. To provide an overview of this osteoimmune imbalance, **Figure 1** summarizes how activated T cells reshape the bone microenvironment and shift bone remodeling toward excessive resorption.

## 2 Physiological basis by which T-cell subsets maintain bone metabolic homeostasis

### 2.1 The interaction network between T cells and bone cells

Bone marrow is not only a hematopoietic organ but also a niche composed of immune cells, osteocytes and stromal cells. T cells are not mere bystanders in the bone marrow; rather, they participate in bone remodeling via soluble cytokines, cell-cell contact, and metabolic products. When RANKL is expressed, activated T cells can directly promote the differentiation of monocyte/macrophage lineage precursors into osteoclasts (22); in parallel, TNF-α, IL-17, IL-6 and GM-CSF released by T cells can enhance the recruitment and maturation of osteoclast precursors, whereas signals such as IL-4, IL-10, TGF-β and CTLA-4 can inhibit this process (23).

Osteoblasts and bone marrow mesenchymal stem cells are also regulated by T cells. Under certain conditions, IL-17 released by Th17 cells can inhibit osteoblast mineralization or induce osteoblasts to express RANKL, thereby indirectly promoting osteoclastogenesis (24, 25); IL-10 and TGF-β derived from Tregs can reduce levels of inflammatory cytokines and improve the microenvironment for osteogenic differentiation of BMSCs; IL-4 and IL-13 derived from Th2 cells can inhibit RANKL-induced osteoclast fusion and may protect osteoblast function through anti-inflammatory effects (26–29). Consequently, T-cell subsets cannot be simply categorized as “proresorptive” or “pro-osteogenic”, but rather influence bone resorption, bone formation and their coupling through complex combinations of cytokines.

As the most abundant and longest-lived cells in bone tissue, osteocytes also play a significant role in T-cell-mediated regulation of bone metabolism (30). Osteocytes express RANKL and participate in osteoclast recruitment, while secreting sclerostin to inhibit the Wnt/β-catenin osteogenic signaling pathway (31). Under chronic inflammatory conditions, T-cell-derived TNF-α, IL-17 and IFN-γ may alter the RANKL/OPG ratio and sclerostin expression in osteocytes, transforming them from mechanosensors and maintainers of bone homeostasis into amplifiers of inflammatory bone resorption. Therefore, antiosteoporotic regulation by T-cell subsets requires consideration not only of the immune cells themselves, but also of the multicellular communication between them and osteocytes, stromal cells and vascular endothelial cells.

### 2.2 Transcriptional regulation of CD4+ T-cell differentiation

Naive CD4+ T-cell differentiation is determined by a combination of antigen stimulation, costimulatory signals, cytokines and metabolic status. Th1 differentiation is driven by IL-12/STAT4, IFN-γ/STAT1 and T-bet; Th2 differentiation depends on IL-4/STAT6 and GATA-3 (32);Th17 differentiation requires TGF-β in conjunction with IL-6, IL-1β, IL-21 or IL-23, with RORγt serving as the core transcription factor; Tregs differentiation is closely associated with IL-2/STAT5 and Foxp3 (33). Competition and mutual inhibition exist between these transcription factors; for example, Foxp3 can restrict RORγt function, whereas T-bet and GATA-3 stabilize the Th1 and Th2 lineages, respectively. The chronic inflammatory milieu associated with OP often elevates levels of IL-6, TNF-α and IL-1β, thereby making CD4+ T cells more prone to adopt Th17 or Th1 inflammatory phenotypes.

### 2.3 Costimulatory molecules and immune checkpoints

T-cell activation requires TCR recognition of peptide-MHC complexes, as well as costimulation via CD28 and CD80/CD86. Excessive costimulation can sustain effector T-cell activation and increase the release of RANKL, TNF-α and IL-17 (34); conversely, negative regulatory molecules such as CTLA-4 and PD-1 can limit excessive T-cell responses and maintain immune tolerance (35). CTLA-4 is not only a key molecule in the immunosuppressive function of Tregs, but also directly inhibits osteoclast formation; CD80/CD86 can also negatively regulate osteoclastogenesis via the IDO/tryptophan-metabolism pathway (36, 37). These findings establish the ‘immune checkpoint–osteoclast differentiation’ pathway as one of the key mechanisms in T-cell-based antiosteoporotic therapy.

### 2.4 T-cell metabolism and osteoimmune homeostasis

Upon activation, T cells transition from a quiescent state to proliferative and effector states, which requires reorganization of glycolysis, glutamine metabolism, fatty acid metabolism and mitochondrial oxidative phosphorylation (38, 39). Th17 cells rely predominantly on glycolysis and HIF-1α signaling, whereas Tregs rely more on fatty acid oxidation and mitochondrial homeostasis; mTOR promotes effector T-cell differentiation, whereas AMPK contributes to energy homeostasis and Tregs maintenance (40, 41). Aging, estrogen deficiency and a high-glucose environment can lead to ROS accumulation and mitochondrial dysfunction, thereby maintaining T cells in a pro-inflammatory phenotype. Consequently, metabolic reprogramming is a key intermediate step in T-cell-mediated OP. The major T-cell subsets involved in bone metabolism, together with their representative transcription factors, cytokines and predominant effects on bone remodeling, are summarized in **Table 1**.

**Table 1:** T-cell subsets and their characteristics in regulating bone metabolism.

T-cell subsets	Major transcription factors/markers	Representative cytokines or molecules	Main effects on bone metabolism
Th17	RORγt, STAT3	IL-17A/F, IL-21, IL-22, GM-CSF, RANKL	Promotes RANKL expression and osteoclast differentiation, thereby amplifying inflammatory bone resorption (42, 46)
Tregs	Foxp3, STAT5, CD25	IL-10, TGF-β, CTLA-4, IDO-related signaling	Inhibits osteoclastogenesis, limits inflammation, and maintains bone marrow immune homeostasis (57, 60)
Th1	T-bet, STAT1/4	IFN-γ, TNF-α, IL-2	IFN-γ has a biphasic effect; in chronic inflammation, it may indirectly promote bone resorption (20, 72)
Th2	GATA-3, STAT6	IL-4, IL-13, IL-5	Typically inhibits osteoclast differentiation and attenuates inflammatory bone resorption (76, 77)
CD8+ T	CD8, TCR, aging marker CD57/KLRG1	IFN-γ, TNF-α, granzymes, and Wnt10b in some subpopulations	Aging-like CD8+ T cells promote inflammation and bone loss; some subpopulations may promote bone formation (78, 80)
γδ T	TCRγδ	IL-17, IFN-γ, TNF-α	Rapidly produces IL-17 and is involved in the mucosa-bone axis and inflammatory bone resorption (81, 84)

This table summarizes the major T-cell subsets involved in the regulation of bone metabolism, including their representative transcription factors, surface markers, cytokines, and predominant effects on osteoclastogenesis, osteogenesis, and osteoimmune homeostasis. The biological effects of each subset should be interpreted in a context-dependent manner, as their functions may vary according to disease stage, inflammatory status, tissue microenvironment, and cytokine exposure. Th, T helper cell; Tregs, regulatory T cell; RORγt, retinoic acid receptor-related orphan receptor gamma t; STAT, signal transducer and activator of transcription; Foxp3, forkhead box P3; CD, cluster of differentiation; TCR, T-cell receptor; KLRG1, killer cell lectin-like receptor G1; IL, interleukin; GM-CSF, granulocyte-macrophage colony-stimulating factor; RANKL, receptor activator of nuclear factor-κB ligand; CTLA-4, cytotoxic T-lymphocyte-associated protein 4; IDO, indoleamine 2,3-dioxygenase; IFN-γ, interferon-gamma; TNF-α, tumor necrosis factor-alpha.

## 3 Mechanisms of T-cell-mediated regulation of bone remodeling in osteoporosis

### 3.1 Th17/Tregs imbalance: the central axis of T-cell-mediated osteoporosis regulation

#### 3.1.1 Molecular mechanisms by which Th17 cells promote bone resorption

Th17 cells are among the most extensively studied pro-resorptive T-cell subsets. Their signature cytokine, IL-17A, acts on osteoblasts, bone marrow stromal cells, synovial fibroblasts and immune cells to induce the expression of RANKL, IL-6, TNF-α, PGE2 and chemokines, thereby promoting the recruitment and differentiation of osteoclast precursors (42). In addition to indirectly promoting osteoclastogenesis by increasing RANKL expression in osteoblast-lineage and stromal cells, IL-17A can directly upregulate RANK expression on osteoclast precursors, thereby enhancing their responsiveness to RANKL stimulation (24) N2. Following its binding to the IL-17RA/IL-17RC receptor complex, IL-17A recruits the adaptor protein Act1 and downstream TRAF6, leading to the activation of NF-κB and MAPK signaling pathways (43–45). IL-17A and TNF-α exhibit synergistic osteoclastogenic effects by reinforcing NF-κB and MAPK activation. Moreover, IL-17A–IL-17RA/IL-17RC–Act1–TRAF6 signaling converges with RANKL–RANK–TRAF6 signaling, resulting in sustained activation of c-Fos and NFATc1 and increased expression of osteoclast-associated genes, including TRAP, CTSK, MMP-9 and DC-STAMP (44, 46–49). In postmenopausal osteoporosis and inflammatory bone destruction, Th17 cells act both as effectors of the inflammatory response and as amplifiers of RANKL expression.

Th17 differentiation is regulated by IL-6, IL-1β, TGF-β, IL-21 and IL-23, with the STAT3-RORγt axis being the most critical (33, 43). Estrogen deficiency can activate antigen-presenting cells and elevate levels of IL-6, IL-23 and TNF-α, thereby promoting Th17 expansion. Animal studies have shown that ovariectomy increases the differentiation and expansion of IL-17-producing T cells, whereas IL-17 neutralization or disruption of IL-17 receptor-associated signaling attenuates osteoclastogenesis and estrogen-deficiency-induced bone loss (44, 50, 51). Act1, an adaptor protein downstream of the IL-17 receptor, is also involved in estrogen-deprivation-induced bone loss, indicating that the IL-17–Act1 axis is a key pathway mediating the osteoclastic effects of Th17 cells (44, 45, 50, 51). Furthermore, PTH-induced bone resorption is also associated with increased Th17 and IL-17 signaling, indicating that Th17 cells are not only involved in postmenopausal osteoporosis but may also contribute to bone loss associated with parathyroid hormone abnormalities (52, 53).

Recent pharmacological evidence further supports the involvement of the Th17/IL-17/NF-κB pathway in estrogen-deficiency-induced bone loss. Zhang et al. reported that ovariectomy enhanced the Th17 response and increased the expression of CD4, RORγt, IL-17A and RANKL, accompanied by increased NF-κB p65 phosphorylation and elevated expression of the osteoclastogenic molecules NFATc1 and CTSK. Treatment with Yougui pills suppressed these alterations and reduced osteoclastogenesis and bone loss, whereas exogenous IL-17 enhanced osteoclast formation and bone-resorptive activity (54). These findings provide additional evidence that inhibition of the Th17/IL-17/NF-κB/NFATc1 axis can attenuate osteoclast activation, although the results should be interpreted as pharmacological evidence rather than definitive proof of a single causal pathway. Clinical observations showing positive associations of IL-17A with soluble RANKL and the RANKL/OPG ratio, as well as an inverse association with lumbar bone mineral density, further support the relevance of this pathway in postmenopausal women (55, 56).

#### 3.1.2 Bone-protective mechanisms of Tregs cells

Tregs cells, characterized primarily by Foxp3 expression, can suppress immune inflammation and protect bone mass via soluble factors and contact-dependent mechanisms (57–59). In terms of osteoclast regulation, Tregs can inhibit the differentiation of monocytes into osteoclasts, reduce the formation of TRAP-positive multinucleated cells, and decrease osteoclastic resorption pits (60). The mechanisms involved include the secretion of anti-inflammatory factors such as IL-10 and TGF-β, which inhibit the production of RANKL and TNF-α by pro-inflammatory cells; more importantly, Tregs express CTLA-4, which can bind to CD80/CD86 on the surface of osteoclast precursors or antigen-presenting cells, thereby directly inhibiting osteoclastogenesis or inducing the IDO pathway (61, 62).

Tregs also exert an indirect protective effect on osteoblasts and BMSCs. Chronic inflammation inhibits the osteogenic differentiation of BMSCs and promotes their adipogenic differentiation; by reducing the levels of TNF-α, IL-1β and IL-6, Tregs provide a relatively stable microenvironment for the expression of Runx2, Osterix, ALP, COL1A1 and OCN (63). Tregs may also regulate osteoimmune-osteogenic coupling via Wnt10b, TGF-β and cell-cell contact. Notably, TGF-β plays a crucial role in both Tregs induction and bone matrix remodeling, but its effects are concentration- and time-dependent; excessive or persistent TGF-β signaling may lead to abnormal mineralization or fibrotic-like repair (55). Therefore, Tregs-targeted therapy should aim to restore homeostasis rather than indiscriminately intensify immunosuppression.

#### 3.1.3 Regulatory determinants and clinical significance of the Th17/Tregs balance

Clinical studies have reported an increased frequency of Th17 cells and elevated IL-17 levels in postmenopausal women with low bone mineral density, whereas changes in circulating Tregs numbers have been less consistent across studies (55). Therefore, the Th17/Tregs ratio should be interpreted together with the functional status of both cell subsets rather than being regarded solely as a numerical indicator. The Th17/Tregs balance is regulated by multiple interacting factors. At the cytokine and transcriptional levels, IL-6, IL-1β, IL-21 and IL-23 activate the STAT3–RORγt program and promote Th17 differentiation, whereas IL-2–STAT5 signaling supports Foxp3 expression and the survival and stability of Tregs (32, 33). TGF-β exerts context-dependent effects: in the presence of IL-2 and a relatively non-inflammatory environment, it favors Tregs differentiation, whereas its combination with IL-6 or IL-1β promotes Th17 polarization. Estrogen deficiency further shifts the balance toward Th17 dominance by enhancing antigen-presenting-cell activation and inflammatory cytokine production while weakening Tregs-mediated immune suppression (26, 50).

Cellular metabolism also influences the lineage fate of CD4+ T cells. HIF-1α-dependent glycolysis and mTOR activity favor Th17 differentiation, whereas mitochondrial oxidative phosphorylation, fatty-acid oxidation and metabolic homeostasis contribute to Tregs maintenance (40, 41). Accordingly, hypoxia, oxidative stress, hyperglycemia and mitochondrial dysfunction may increase the Th17/Tregs ratio by promoting an inflammatory metabolic phenotype. Environmental and intestinal factors provide an additional regulatory layer. High extracellular sodium can promote pathogenic Th17 differentiation through p38/MAPK–NFAT5–SGK1 and SGK1–IL-23R signaling (64, 65). In contrast, microbiota-derived butyrate can expand intestinal and bone-marrow Tregs and promote Treg-dependent Wnt10b production, whereas segmented filamentous bacteria favor intestinal Th17 differentiation (66, 67).

A persistent shift toward Th17 dominance has multiple pathological consequences for bone remodeling. Increased Th17 activity elevates IL-17A, TNF-α and RANKL production, increases the RANKL/OPG ratio and reinforces NF-κB/MAPK/NFATc1-dependent osteoclast differentiation and bone-resorptive activity (18, 19). Concurrent Tregs insufficiency or functional instability weakens the inhibitory effects mediated by IL-10, TGF-β, CTLA-4 and IDO, thereby allowing inflammatory T-cell and osteoclastogenic signals to persist (57, 59). In addition to stimulating bone resorption, prolonged exposure to IL-17 and TNF-α may impair BMSC osteogenic differentiation and suppress osteoblast-associated programs, resulting in the uncoupling of bone resorption from bone formation (25, 63). Collectively, these changes promote excessive bone turnover, trabecular microarchitectural deterioration, progressive loss of bone mass and increased susceptibility to fragility fractures.

Although clinical studies have associated increased Th17 frequency and IL-17 levels with reduced bone mineral density, a standardized pathological threshold for the Th17/Tregs ratio has not yet been established (55). Future studies should therefore evaluate this ratio together with serum IL-17A, RANKL/OPG, TNF-α, CTX, P1NP and DXA or HR-pQCT parameters, which may facilitate the identification of patients with inflammation-dominant osteoimmune phenotypes.

#### 3.1.4 Antiosteoporotic strategies targeting Th17/Tregs

**Figure 2:**
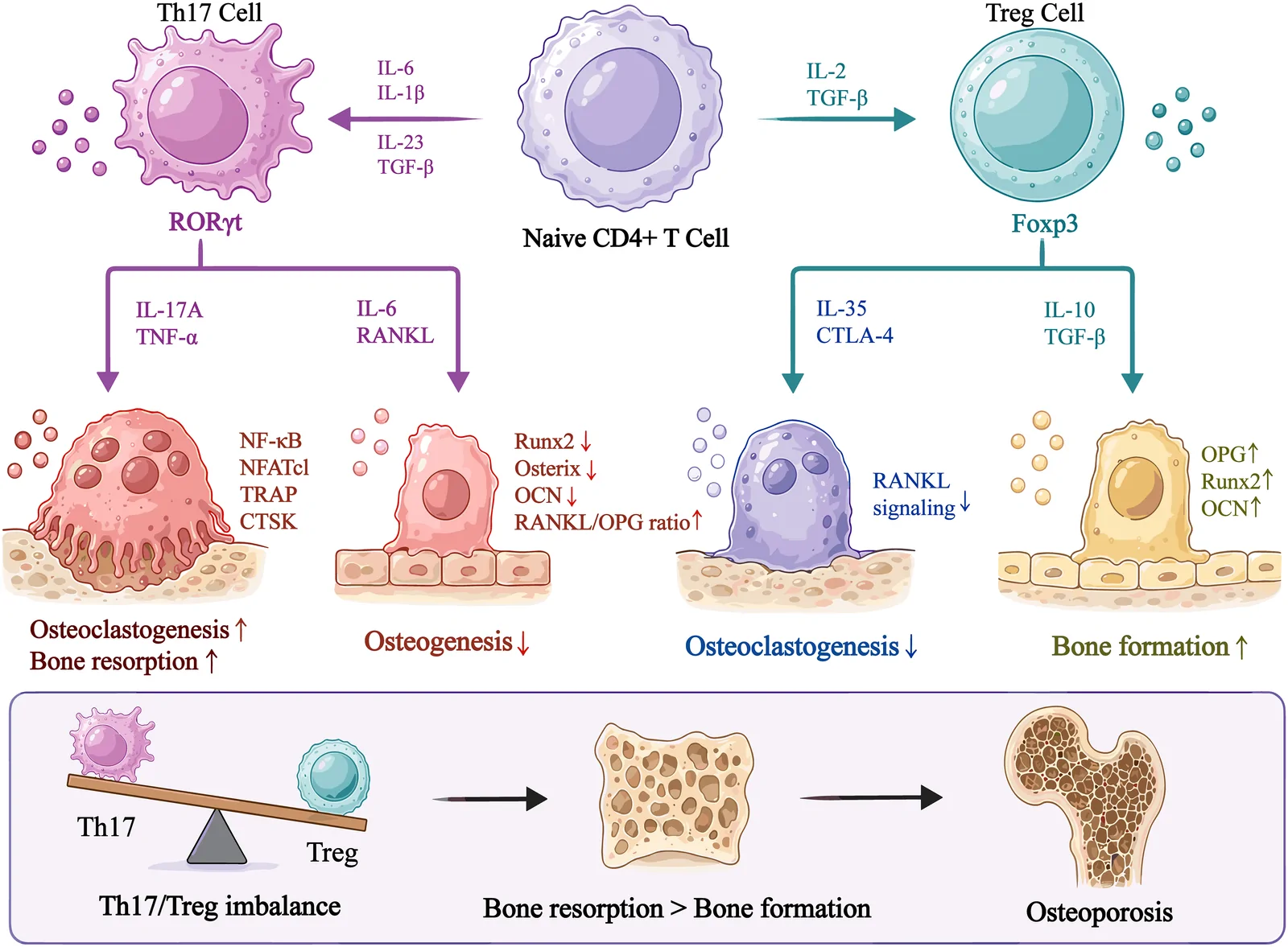
Th17/Tregs imbalance as a central osteoimmune mechanism in osteoporosis. Naive CD4+ T cells differentiate into Th17 cells under the stimulation of IL-6, IL-1β, IL-23 and TGF-β, whereas IL-2 and TGF-β favor Tregs differentiation. Th17 cells, characterized by RORγt expression, promote osteoclastogenesis and bone resorption by producing IL-17A, TNF-α, IL-6 and RANKL. These mediators activate osteoclastogenic signaling pathways, including NF-κB, NFATc1, TRAP and CTSK, and suppress osteogenic activity by reducing Runx2, Osterix and OCN expression while increasing the RANKL/OPG ratio. In contrast, Foxp3+ Tregs cells inhibit osteoclastogenesis and support bone formation through IL-10, TGF-β, IL-35 and CTLA-4. A shift toward Th17 dominance and Tregs insufficiency disrupts osteoimmune homeostasis, resulting in bone resorption exceeding bone formation and contributing to osteoporosis. Th17, T helper 17 cell; Tregs, regulatory T cell; IL, interleukin; TGF-β, transforming growth factor-beta; RORγt, retinoic acid receptor-related orphan receptor gamma t; Foxp3, forkhead box P3; TNF-α, tumor necrosis factor-alpha; RANKL, receptor activator of nuclear factor-κB ligand; OPG, osteoprotegerin; NF-κB, nuclear factor-kappa B; NFATc1, nuclear factor of activated T cells cytoplasmic 1; TRAP, tartrate-resistant acid phosphatase; CTSK, cathepsin K; Runx2, runt-related transcription factor 2; OCN, osteocalcin; CTLA-4, cytotoxic T-lymphocyte-associated protein 4.

In principle, inhibiting IL-17, IL-6/STAT3 and RORγt, or promoting Foxp3, IL-2/STAT5 and CTLA-4, may all improve the osteoimmune microenvironment in OP (68, 69). However, the direct use of potent immunosuppressants to treat generalized OP is not practical, as osteoporosis is predominantly a long-term chronic condition requiring treatment that balances anti-infective and anti-tumor immune responses with long-term safety. A more reasonable strategy may be to select patient groups with a clear inflammatory phenotype—such as those with concomitant autoimmune diseases, inflammatory bowel disease, periodontitis, diabetes, or significant Th17/Tregs imbalance—and apply stratified immunomodulatory interventions. For patients with general osteoporosis, the Th17/Tregs balance can be improved through exercise, nutrition, vitamin D, probiotics or mild immunometabolic modulation. The opposing effects of Th17 cells and Tregs on osteoclastogenesis, osteogenesis and osteoimmune homeostasis are schematically shown in **Figure 2**.

### 3.2 Mechanisms of action of other T-cell subsets in osteoporosis

#### 3.2.1 Th1/Th2 balance and the biphasic effects of IFN-γ

Th1 cells mainly secrete IFN-γ, IL-2 and TNF-α, whereas Th2 cells mainly secrete IL-4, IL-5 and IL-13 (70, 71). IFN-γ exerts pronounced biphasic effects on bone metabolism: *in vitro*, IFN-γ induces the degradation of TRAF6, thereby inhibiting RANKL-mediated osteoclast differentiation; however, *in vivo* under conditions of estrogen deficiency or chronic inflammation, IFN-γ can enhance the function of antigen-presenting cells and T-cell activation, promoting the production of TNF-α and RANKL by T cells, ultimately producing a pro-resorptive effect (72–75). Therefore, IFN-γ is neither strictly antiresorptive nor pro-resorptive; its effects depend on the immune environment and the cellular origin.

Th2 cells and their cytokines, IL-4 and IL-13, generally exert antiresorptive effects. IL-4 activates STAT6 and inhibits NF-κB-related signaling, thereby reducing RANKL- or TNF-α-induced osteoclast formation (76). IL-13 can also mitigate inflammatory bone resorption by inhibiting osteoclast precursor fusion and regulating macrophage polarization (77). In the treatment of osteoporosis, reducing pathological Th1/TNF-α signaling whereas preserving or enhancing moderate Th2 anti-inflammatory signaling may help restore the balance of bone remodeling. However, an excessive shift toward Th2 may also lead to problems such as hypersensitivity or fibrosis; therefore, precise regulation is equally necessary.

#### 3.2.2 CD8+ T cells and immunological aging

CD8+ T cells participate in osteoimmune regulation via IFN-γ, TNF-α, granzymes and membrane-bound RANKL. With advancing age, thymic atrophy, chronic infections, repeated antigenic stimulation and metabolic stress can lead to terminal differentiation or an aging-like phenotype in CD8+ T cells, characterized by CD28 loss, elevated expression of KLRG1 and CD57, and increased secretion of TNF-α, IFN-γ and cytotoxic molecules (78, 79). These senescent-like CD8+ T cells may contribute to an inflammaging milieu by increasing TNF-α, IFN-γ and cytotoxic mediators, thereby impairing BMSC osteogenic potential and promoting osteoclast activation, both of which are associated with age-related bone loss.

The role of CD8+ T cells in bone formation remains incompletely resolved. On one hand, certain CD8+ T cell subsets can release osteogenic factors such as Wnt10b and participate in intermittent PTH-induced bone formation responses (66); on the other hand, aged or persistently activated CD8+ T cells can accelerate bone loss via pro-inflammatory factors (80). Therefore, future research needs to distinguish between protective CD8+ T cells and pathologically aged CD8+ T cells; CD8+ T cells should not simply be regarded as a single therapeutic target for suppression. Single-cell sequencing, TCR clonality profiles and spatial transcriptomics can help identify the status of CD8+ T cells in different subtypes of OP.

#### 3.2.3 γδ T cells and unconventional T cells

γδ T cells have features of both innate and adaptive immunity and can rapidly produce IL-17, IFN-γ or TNF-α during early inflammation (81).γδ T17 cells are a major source of IL-17, promoting RANKL expression and osteoclast formation; however, in the defense against infection and tissue repair, γδ T cells may also exert a protective role via IFN-γ or tissue repair factors (82–84). Consequently, the role of γδ T cells in osteoporosis depends on their subpopulation, tissue localization and stage of activation. In chronic intestinal inflammation, periodontitis or autoimmune diseases, the sustained activation of γδ T17 cells may be a major driver of bone loss.

Unconventional T cells, such as NKT cells and MAIT cells, are also increasingly attracting attention. Characterized by semi-invariant TCRs, tissue residency and rapid cytokine release, they participate in mucosal immunity and tissue repair via IFN-γ, TNF-α, IL-17 or IL-22 (85, 86). Although direct evidence regarding their role in osteoporosis (OP) remains limited, the influence of the gut microbiota, bile acid metabolism and mucosal immunity on bone mass suggests that unconventional T cells may be potential regulatory nodes within the gut-immune-bone axis. Their role could be further validated in models of gut microbiota alterations, age-related OP and diabetic bone disease.

#### 3.2.4 Tfh, Th9 and Th22 cells

**Figure 3:**
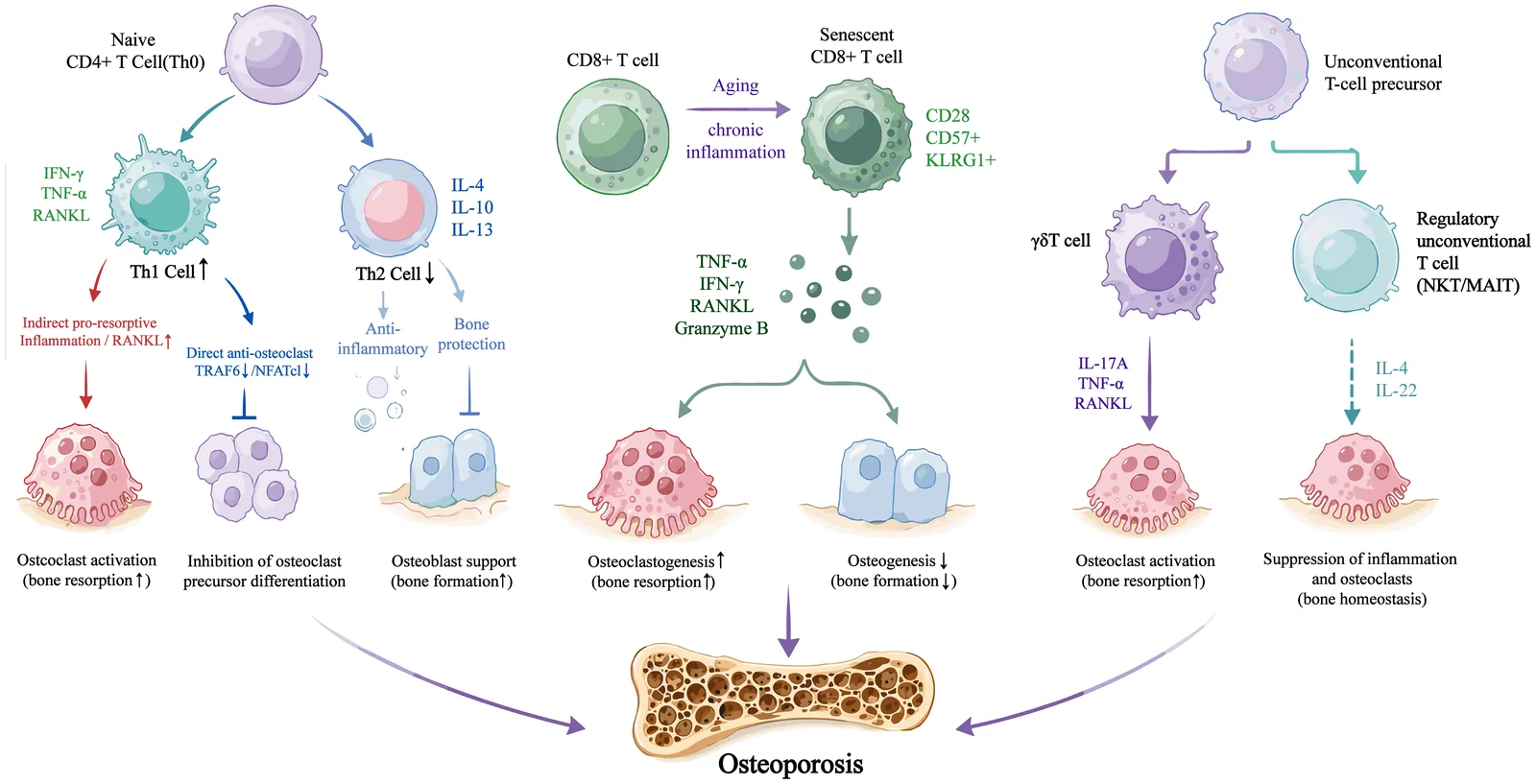
Regulatory effects of Th1/Th2 cells, CD8+ T cells and unconventional T cells on osteoporotic bone remodeling. Multiple T-cell subsets participate in osteoimmune regulation beyond the classical Th17/Tregs axis. Th1 cells mainly secrete IFN-γ, TNF-α and RANKL. IFN-γ may directly suppress osteoclast precursor differentiation by inhibiting TRAF6/NFATc1 signaling, but under chronic inflammatory conditions, Th1-associated cytokines can indirectly promote osteoclast activation and bone resorption. Th2 cells secrete IL-4, IL-5 and IL-13, which generally exert anti-inflammatory and bone-protective effects by inhibiting osteoclastogenesis and supporting osteoblast function. During aging and chronic inflammation, CD8+ T cells may acquire a senescent phenotype characterized by CD28 loss and increased CD57/KLRG1 expression, producing TNF-α, IFN-γ, RANKL and granzyme B, thereby enhancing osteoclastogenesis and suppressing osteogenesis. γδ T cells can rapidly release IL-17A, TNF-α and RANKL to promote osteoclast activation, whereas regulatory unconventional T cells, including NKT and MAIT cells, may contribute to immune restraint and bone homeostasis through anti-inflammatory cytokines such as IL-4 and IL-22. The combined effects of these subsets converge on excessive bone resorption, insufficient bone formation and osteoporosis. Th1, T helper 1 cell; Th2, T helper 2 cell; IFN-γ, interferon-gamma; TNF-α, tumor necrosis factor-alpha; RANKL, receptor activator of nuclear factor-κB ligand; TRAF6, tumor necrosis factor receptor-associated factor 6; NFATc1, nuclear factor of activated T cells cytoplasmic 1; IL, interleukin; CD, cluster of differentiation; KLRG1, killer cell lectin-like receptor G1; γδ T cell, gamma delta T cell; NKT cell, natural killer T cell; MAIT cell, mucosa-associated invariant T cell.

Tfh cells regulate B-cell maturation, antibody production and the balance of B-cell-derived OPG/RANKL via signaling pathways such as IL-21 and ICOS, and may thus indirectly influence bone metabolism (87). Th22 cells release IL-22 and possess dual functions in epithelial repair and inflammation regulation; they may influence bone marrow inflammation via the intestinal barrier and mucosal immunity. Th9 cells produce IL-9 and play an important role in allergic, autoimmune and tumor immunity, but their significance for bone metabolism remains unclear (88).Although these subpopulations have not yet been integrated into a mature mechanistic pathway within the field of bone biology, they help to explain why patients with the same degree of bone density loss may exhibit different immune phenotypes and treatment responses. In addition to the classical Th17/Treg axis, other T-cell subsets also contribute to osteoporotic bone remodeling through context-dependent pro-resorptive or protective mechanisms, as illustrated in **Figure 3**.

## 4 Differences in T-cell mechanisms across different types of osteoporosis

### 4.1 Postmenopausal osteoporosis

The major etiologic factor in postmenopausal OP is a decline in estrogen levels. Estrogen not only acts directly on osteoblasts, osteoclasts and osteocytes, but also regulates bone metabolism via the immune system (89). Estrogen deficiency can enhance the activation of antigen-presenting cells, increase signaling of IL-7, IL-12, IL-18, TNF-α and IFN-γ, and promote T-cell expansion and prolonged survival (90–92). Concurrently, estrogen deficiency can induce Th17 cell differentiation and an increase in IL-17 (93);neutralization of IL-17 or blockade of IL-17 signaling can attenuate OVX-induced bone loss (94). This suggests that postmenopausal osteoporosis (OP) is not only an endocrine disorder but also a classic bone-immune disease. Human evidence further indicates that postmenopausal women with osteoporosis exhibit increased T-cell production of osteoclastogenic cytokines and greater osteoclastogenic activity than postmenopausal women with normal bone mass, supporting a direct association between estrogen deficiency, T-cell activation and enhanced bone resorption (95).

In postmenopausal OP, impaired Tregs function is equally significant. Estrogen enhances the ability of Tregs to suppress osteoclast differentiation and bone resorption (26), whereas estrogen deficiency weakens immune tolerance, leading to an increased Th17/Tregs ratio (60). Clinical studies have reported increased Th17-cell frequencies and elevated IL-17 production in postmenopausal women with osteoporosis, although changes in circulating Tregs numbers are not entirely consistent among cohorts (96). These findings suggest that both the numerical Th17/Tregs ratio and the functional status of the two subsets should be considered when characterizing the female osteoimmune phenotype (55). Clinically, if patients present with markedly elevated inflammatory markers, intestinal inflammation or an autoimmune background, T-cell-related targets may be of greater value for intervention.

This osteoimmune profile differs from that of age-matched men. In women, the menopausal transition causes an abrupt decline in estrogen, promoting Th17 polarization, weakening Treg-mediated control, and increasing TNF-α-, IL-17-, and RANKL-dependent osteoclastogenesis (50) N7. In men, sex-steroid decline is generally more gradual, and estradiol deficiency appears to be a major driver of increased bone resorption (97). Sex-specific immune aging may further contribute, as men show a stronger age-related shift from adaptive toward innate and pro-inflammatory immune activity (98). However, direct comparisons of T-cell subsets between male and female patients with osteoporosis remain limited.

### 4.2 Senile osteoporosis

Senile osteoporosis is closely associated with immunological aging. With advancing age, thymic output decreases, the naive T-cell pool shrinks, and there is an increase in memory/effector T cells and senescent-like T cells, alongside the persistence of chronic low-grade inflammation (99–101). Aging T cells can produce TNF-α, IFN-γ and other inflammatory mediators, promoting osteoclast activation and inhibiting the osteogenic differentiation of BMSCs. Concurrently, myeloid fat accumulation and impaired mitochondrial function further deplete the supply of osteoblasts, leading to insufficient bone formation. Senile osteoporosis therefore often manifests as a coexistence of low bone formation and inflammatory bone resorption.

T-cell regulation in senile OP should emphasize the reversal of immunosenescence rather than simple anti-inflammatory therapy. Potential approaches include improving mitochondrial function, reducing ROS, modulating mTOR/AMPK, restoring the homeostasis of naive T cells and Tregs, improving the gut microbiota, and increasing the production of short-chain fatty acids (102). Compared with postmenopausal osteoporosis, age-related osteoporosis exhibits greater immunological heterogeneity, and the blockade of a single cytokine may not be sufficient; a multidimensional assessment of T-cell aging markers, inflammatory factors and bone turnover indices may facilitate refined subtyping.

### 4.3 Diabetic osteoporosis

Diabetes can affect bone quality through hyperglycemia, abnormal insulin/IGF-1 signaling, advanced glycation end products (AGEs), oxidative stress and chronic inflammation. A hyperglycemic environment can promote T-cell metabolic abnormalities, enhance the pro-inflammatory bias of Th17 and Th1 cells, whereas simultaneously impairing Tregs function and mitochondrial homeostasis (103). AGEs-RAGE signaling can also activate antigen-presenting cells and the NF-κB pathway, indirectly promoting the production of TNF-α and IL-17 by T cells (104). The increased risk of fractures in diabetic patients is not fully explained by bone mineral density; bone matrix quality, microvascular lesions and immune and inflammatory processes all play a role.

### 4.4 Glucocorticoid-induced and inflammatory osteoporosis

Long-term glucocorticoid use can suppress osteogenesis, promote osteocyte apoptosis and alter T-cell and macrophage function (105).Although glucocorticoids have immunosuppressive effects, long-term use can also result in reduced bone formation and a compromised bone marrow microenvironment (106, 107). Bone loss associated with inflammatory bowel disease, rheumatoid arthritis, systemic lupus erythematosus and periodontitis more directly reflects the osteoclast-promoting role of T cells: sustained upregulation of Th17, TNF-α, IL-6 and RANKL signaling, coupled with insufficient Tregs function, leads to increased local or systemic bone resorption (108–110). These conditions provide important clinical scenarios for T-cell-targeted therapies against bone loss.

### 4.5 Disuse and microgravity-associated bone loss

**Figure 4:**
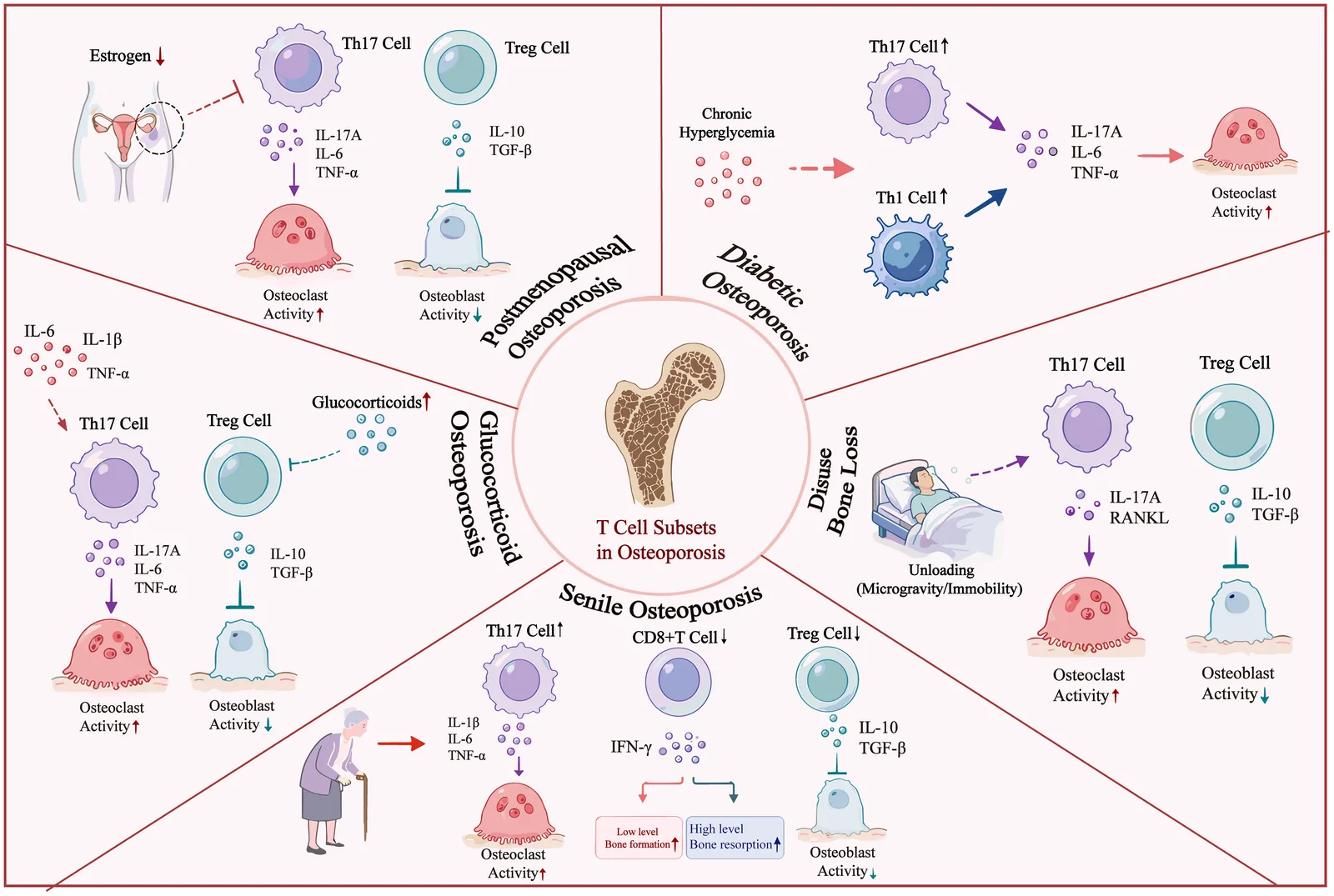
T-cell subset dysregulation across different types of osteoporosis. Different etiological forms of osteoporosis share a common osteoimmune feature: dysregulated T-cell responses disturb the balance between osteoclast-mediated bone resorption and osteoblast-mediated bone formation. In postmenopausal osteoporosis, estrogen deficiency promotes Th17 expansion, increases IL-17A, IL-6 and TNF-α production, and weakens Tregs-mediated immune suppression, leading to enhanced osteoclast activity and reduced osteoblast function. In diabetic osteoporosis, chronic hyperglycemia and metabolic inflammation promote Th17/Th1 polarization and pro-inflammatory cytokine release, thereby increasing osteoclast activity. In glucocorticoid-induced osteoporosis, inflammatory cytokines such as IL-6, IL-1β and TNF-α, together with glucocorticoid-related suppression of osteogenesis, favor Th17 activation, Tregs dysfunction and impaired osteoblast activity. In senile osteoporosis, immunosenescence is characterized by increased Th17 activity, reduced Tregs function and altered CD8+ T-cell responses, contributing to both enhanced bone resorption and reduced bone formation. In disuse-related bone loss, unloading or immobilization alters T-cell-associated inflammatory signals, increases IL-17A and RANKL-mediated osteoclast activation, and suppresses osteoblast activity. Collectively, these disease-specific T-cell abnormalities converge on osteoimmune imbalance and progressive osteoporotic bone loss. IL, interleukin; TNF-α, tumor necrosis factor-alpha; TGF-β, transforming growth factor-beta; Th1, T helper 1 cell; Th17, T helper 17 cell; Tregs, regulatory T cell; IFN-γ, interferon-gamma; RANKL, receptor activator of nuclear factor-κB ligand; CD8+ T cell, cluster of differentiation 8-positive T cell.

During prolonged bed rest, immobilization or exposure to microgravity, the mechanical sensitivity of osteocytes decreases, sclerostin levels in compact bone rise, and osteogenic signaling is attenuated (111). The immune system may also be affected by reduced physical activity, muscle atrophy and metabolic changes, leading to alterations in T-cell cytokines. Although the core mechanism of disuse-related bone loss is insufficient mechanical loading, low-grade inflammation and immunometabolic changes may influence its progression (112). Exercise intervention can both increase mechanical loading on the bones and improve the inflammatory phenotype of T cells and the gut microbiota, representing an important non-pharmacological strategy linking mechanical stimulation with immune regulation. Although different types of osteoporosis have distinct etiological backgrounds, their T-cell-related abnormalities converge on disturbed osteoimmune homeostasis, as summarized in **Figure 4**. To facilitate comparison among different forms of osteoporosis, **Table 2** summarizes their major T-cell abnormalities, mechanistic characteristics and potential therapeutic implications.

**Table 2:** Characteristics of T-cell abnormalities in different types of osteoporosis.

Type of Osteoporosis	Major T-cell abnormalities	Mechanistic characteristics	Therapeutic implications
Postmenopausal Osteoporosis	Elevated Th17, reduced Tregs, increased TNF+ T cells	Estrogen deficiency enhances antigen presentation and gut-derived T-cell migration (89, 93)	Focus on Th17/Tregs, the intestinal barrier and RANKL signaling (92, 94)
Age-related Osteoporosis	Increased senescent-like CD8+ T cells and memory T cells	Co-occurrence of inflammatory aging, mitochondrial damage and impaired osteogenesis (99, 102)	Immunometabolic and anti-aging interventions (101, 102)
Diabetic Osteoporosis	Enhanced Th17/Th1 bias, impaired Tregs function	Driven by high glucose levels, AGEs-RAGE and oxidative stress (103, 104)	Glycemic control, antioxidant therapy and immunometabolic regulation (103, 104)
Glucocorticoid-induced osteoporosis	Glucocorticoid-mediated suppression and dysregulation of T-cell responses	Suppressed osteoblastogenesis, increased osteocyte apoptosis, and altered immune-cell function compromise the bone marrow microenvironment (105, 106)	Minimize glucocorticoid exposure and implement guideline-based bone protection (105, 106)
Inflammatory Osteoporosis	Activated Th17, TNF+ T, and RANKL+ T cells	Local inflammation directly induces osteoclast formation (107, 110)	Control of primary inflammation and preservation of bone mass (107, 110)
Disuse-related Osteoporosis	Inflammatory phenotypes of T cells may be altered	Combined effects of reduced mechanical loading and immunometabolic changes (111, 112)	Exercise combined with inflammation management (119, 120)

This table summarizes representative T-cell abnormalities, major pathogenic features, and potential therapeutic implications across common osteoporosis subtypes. These immune phenotypes may overlap according to age, hormonal status, metabolic disturbance, inflammation, and treatment exposure. Therapeutic implications are mechanistically informed and do not necessarily indicate established clinical efficacy. Th17, T helper 17 cells; Tregs, regulatory T cells; TNF, tumor necrosis factor; RANKL, receptor activator of nuclear factor-κB ligand; AGEs, advanced glycation end products; RAGE, receptor for AGEs.

## 5 Therapeutic strategies for osteoporosis using regulation of T-cell subsets

### 5.1 Inhibition of pro-inflammatory T-cell signaling

Drugs targeting pathways such as Th17/IL-17, TNF-α, IL-6/JAK/STAT and RANKL have been used or are being explored in the treatment of autoimmune diseases or osteoporosis (110). Among these, denosumab is the clinically approved RANKL-neutralizing antiresorptive agent that directly inhibits osteoclast formation, whereas bisphosphonates, SERMs and other antiresorptive therapies also have established clinical roles in osteoporosis management; anti-TNF, anti-IL-6 and anti-IL-17 agents are primarily used for inflammatory diseases, and their improvements in bone density and bone erosion are largely related to the control of inflammation (113, 114). For primary osteoporosis in the general population, the need for direct blockade of IL-17 or TNF-α should be approached with caution, as long-term immunosuppression may increase the risk of infection.

### 5.2 Enhancing Tregs and immune tolerance

Strategies for enhancing Tregs include low-dose IL-2, adoptive transfer of Tregs, expansion of inducible Tregs, CTLA-4-Ig, and promotion of IDO/tryptophan metabolism (61, 115). Animal studies have shown that Tregs can inhibit osteoclast formation and bone resorption; however, clinical application still faces challenges regarding dosage, safety, targeting and the risks associated with immunosuppression. Compared with potent systemic immunomodulatory interventions, local delivery, bone-targeted delivery or short-term use in patients with a highly inflammatory phenotype may be better suited to the long-term management of OP.

### 5.3 Interventions targeting the gut microbiota–T cell–bone axis

The gut microbiota can modulate bone mass (91); its depletion or alteration affects the state of bone marrow immune cells and osteoclast precursors (67). Following estrogen deficiency-induced intestinal barrier damage, gut-derived inflammatory T cells contribute to bone loss (51, 116).Mechanistically, estrogen deficiency-associated dysbiosis and intestinal barrier dysfunction increase microbial antigen exposure, thereby promoting intestinal Th17 and TNF+ T-cell expansion. These inflammatory T cells subsequently migrate to the bone marrow, where IL-17, TNF-α and RANKL enhance osteoclastogenesis and bone loss (51, 117). Conversely, microbiota-derived short-chain fatty acids promote Tregs-mediated immune tolerance; butyrate also stimulates Treg-dependent Wnt10b production by CD8+ T cells, whereas propionate and butyrate directly inhibit osteoclast differentiation by suppressing TRAF6 and NFATc1 signaling (66, 118). Probiotics, prebiotics, dietary fiber and short-chain fatty acids can protect bone mass by promoting Tregs, reducing Th17 cells or modulating bone marrow inflammation (92). The advantage of this approach lies in its high safety profile and suitability for long-term intervention; however, differences in bacterial strains, dosage, gender, age and baseline microbiota can significantly affect efficacy.

### 5.4 Immunometabolic regulation

The metabolic reprogramming of T cells offers a new indirect approach to combating osteoporotic pathogenesis. AMPK activation, moderate mTOR inhibition, improved mitochondrial function, reduced ROS levels and enhanced fatty acid oxidation may all facilitate the transition of T cells from a pro-inflammatory effector state to a Tregs or homeostatic memory state (38). Exercise, energy balance, vitamin D, ω-3 fatty acids, antioxidant nutrients and certain metabolic drugs may influence bone metabolism by improving immunometabolism (119, 120). Future research will need to combine metabolomics, single-cell transcriptomics and functional experiments to clarify which metabolic changes truly mediate the bone-protective effects of T cells.

### 5.5 Combination with existing antiosteoporotic drugs

Conventional antiosteoporotic drugs primarily target bone-resorbing or bone-forming pathways, but may also influence the immune microenvironment. Bisphosphonates act on the monocyte/macrophage–osteoclast lineage and influence γδ T-cell activation (121, 122); PTH-analogues exhibit osteogenic effects when administered intermittently, and some of these effects may be related to T-cell-derived Wnt10b and the bone marrow immune environment (123, 124); romosozumab enhances Wnt signaling by blocking sclerostin (125), which may improve communication between osteocytes and immune cells (126). Future treatments may consider a combined strategy of ‘bone-targeted drugs plus modulation of the immune microenvironment’ to enhance therapeutic efficacy and reduce the risks associated with long-term inhibition of a single signaling pathway.

### 5.6 Cell therapy, exosomes and nanodelivery

Tregs cell therapy, MSC exosomes, bone-targeting nanoparticles and responsive delivery systems provide the technical foundation for the precise regulation of T-cell subsets. An ideal delivery system should be capable of recognizing the inflammatory microenvironment of the bone marrow, releasing anti-inflammatory or Tregs-inducing signals during the active resorption phase, and avoiding excessive immunosuppression during the bone formation phase. Nanomaterials can be surface-modified to achieve bone targeting, T-cell targeting or antigen-presenting cell targeting; however, their long-term safety, immunogenicity, *in vivo* distribution and clinical manufacturability still require systematic evaluation (127).

## 6 Integrated research on key mechanisms and molecular pathways

### 6.1 Mechanistic studies of key molecular pathways

#### 6.1.1 The RANKL/RANK/OPG axis

The RANKL/RANK/OPG axis represents a convergent endpoint through which T cells regulate bone resorption. Th17, Th1 and activated CD8+ T cells can either express RANKL directly or induce osteocytes and stromal cells to express RANKL; conversely, OPG derived from Tregs, Th2 cells and certain B cells can inhibit the binding of RANKL to RANK (128). Under osteoporotic conditions, an elevated RANKL/OPG ratio facilitates the differentiation and maturation of osteoclast precursors, thereby increasing bone resorption (129). Consequently, any antiosteoporotic strategy targeting T-cell subsets should be evaluated for its effects on the RANKL/OPG ratio, TRAF6, NFATc1 and markers of bone resorption.

#### 6.1.2 NF-κB and MAPK pathways

NF-κB is a central pathway in inflammatory responses and osteoclast differentiation. In Th17-mediated osteoclastogenesis, IL-17A binds to the IL-17RA/IL-17RC receptor complex and recruits Act1 and TRAF6, thereby activating NF-κB and MAPK signaling. This pathway not only induces inflammatory and RANKL expression in osteoblast-lineage and stromal cells but also converges with RANKL–RANK–TRAF6 signaling in osteoclast precursors to sustain c-Fos and NFATc1 activation (24, 43, 44, 46) N1. RANKL, TNF-α, IL-1β and IL-17 activate NF-κB through distinct receptor-associated signaling mechanisms and cooperate with MAPK pathways, including JNK, p38 and ERK, to induce NFATc1 and downstream osteoclast-associated genes (130, 131). At the T-cell level, NF-κB is also involved in T-cell activation, cytokine production and survival. Excessive inhibition of NF-κB may impair anti-infectious immunity (131); therefore, a more desirable therapeutic approach is to selectively block the bone marrow inflammatory amplification loop rather than to completely suppress it systemically.

#### 6.1.3 The JAK/STAT pathway

The JAK/STAT pathway determines the differentiation fate of T cells: STAT1 and STAT4 favor Th1, STAT6 favors Th2, STAT3 favors Th17, and STAT5 favors Tregs (132). In OP, enhanced IL-6/STAT3 signaling promotes Th17 differentiation, whereas insufficient IL-2/STAT5 signaling impairs Tregs stability. JAK inhibitors can reduce inflammation and bone erosion in inflammatory diseases, but evidence remains insufficient for their use in primary OP (133). In future, more refined JAK/STAT regulatory strategies could be explored for patients with high osteoimmune inflammation.

#### 6.1.4 PI3K/Akt/mTOR and AMPK

The PI3K/Akt/mTOR pathway regulates T-cell proliferation, effector differentiation and metabolic state; AMPK, meanwhile, senses energy stress and promotes metabolic homeostasis (134). Th17 cells are highly dependent on mTOR/HIF-1α, whereas Tregs rely more on fatty acid oxidation and mitochondrial function. Oxidative stress and metabolic abnormalities in OP can shift T cells toward a pro-inflammatory state (135). Moderate regulation of AMPK/mTOR through exercise, metabolic drugs or nutritional interventions may achieve mild immunometabolic bone protection.

#### 6.1.5 Wnt/BMP/Smad and T-cell–osteogenic coupling

T cells influence not only osteoclasts but also osteoblasts. PTH, Tregs and certain CD8+ T cells can promote osteogenesis via Wnt10b or other signaling pathways; the TGF-β/BMP/Smad and Wnt/β-catenin pathways are key regulatory axes for osteogenic differentiation of bone marrow-derived stem cells (BMSCs) (136). Inflammatory T-cell cytokines such as TNF-α and IL-17 can inhibit osteogenic differentiation or induce osteoblasts to express RANKL. Therefore, immunotherapy for osteoporosis should simultaneously monitor bone formation markers, such as P1NP, BALP, Runx2, OCN and trabecular bone structure, rather than focusing solely on the inhibition of resorption (137). The key T-cell-regulated molecular pathways involved in osteoclastogenesis, effector T-cell differentiation, immune tolerance, immunometabolism and the gut–bone axis are summarized in **Table 3**.

**Table 3:** Key T-cell-regulated pathways in bone metabolism and targets for intervention.

Regulatory level	Key pathways	Key mechanisms	Potential intervention strategies
Osteoclastogenesis	IL-17A–IL-17RA/RC–Act1/TRAF6 and RANKL/RANK–TRAF6–NF-κB/MAPK/NFATc1	Th17-derived IL-17A induces RANKL expression in osteoblast-lineage and stromal cells, upregulates RANK in osteoclast precursors and activates Act1/TRAF6-dependent NF-κB/MAPK signaling, thereby amplifying RANKL-induced NFATc1 activation and osteoclast differentiation (24, 42, 46–48, 54)	Inhibition of Th17 differentiation, IL-17/IL-17 receptor signaling or RANKL/RANK signaling, together with reduction of the RANKL/OPG ratio and pathological NF-κB/NFATc1 activation (128, 140).
Effector T-cell differentiation	IL-6/STAT3/RORγt	An inflammatory environment drives Th17 expansion (43, 48)	Blocking IL-6/STAT3, RORγt or IL-17 signaling (68, 69)
Immune tolerance	IL-2/STAT5/Foxp3, CTLA-4/CD80/86	Enhances Tregs stability and suppresses osteoclast precursor differentiation (57, 62)	Low-dose IL-2, Tregs enhancement, CTLA-4-Ig or local tolerance induction (61, 115)
Immunometabolism	AMPK/mTOR/HIF-1α	Th17 cells favor glycolysis, whereas Tregs favor fatty acid oxidation and mitochondrial homeostasis (38, 41)	Exercise, nutrition, metabolic regulation and antioxidant interventions (119, 120)
Gut microbiota–T-cell–bone axis	Intestinal barrier–microbial metabolites–Th17/Tregs–T-cell trafficking	Estrogen deficiency-associated barrier dysfunction and dysbiosis promote intestinal Th17 and TNF+ T-cell expansion and their migration to the bone marrow, whereas microbial SCFAs and bile acid metabolites regulate Tregs/Th17 differentiation and osteoclastogenic signaling (118, 146).	Probiotics, prebiotics, dietary fiber, SCFA-related interventions, intestinal-barrier restoration and regulation of inflammatory T-cell trafficking (51, 92, 117, 146).

This table summarizes major T-cell-regulated pathways in osteoclastogenesis, immune tolerance, immunometabolism, and the gut–bone axis, together with potential intervention strategies. Most strategies remain mechanistically based or preclinical. IL, interleukin; RANKL/RANK, receptor activator of nuclear factor-κB ligand/receptor; NF-κB, nuclear factor-κB; MAPK, mitogen-activated protein kinase; NFATc1, nuclear factor of activated T cells 1; OPG, osteoprotegerin; STAT, signal transducer and activator of transcription; CTLA-4, cytotoxic T-lymphocyte-associated protein 4; AMPK, AMP-activated protein kinase; mTOR, mechanistic target of rapamycin; HIF-1α, hypoxia-inducible factor-1α; SCFAs, short-chain fatty acids.

### 6.2 Epigenetic, non-coding RNA and spatial microenvironmental mechanisms

#### 6.2.1 Epigenetic regulation is the underlying mechanism governing T-cell lineage stability

Th17 and Tregs cells are not fixed and irreversible states; both exhibit a degree of plasticity in response to changes in inflammation, hypoxia, metabolic stress and the cytokine environment. DNA methylation, histone acetylation and chromatin accessibility at gene loci such as RORC, IL17A, FOXP3, CTLA4 and IL10 determine whether T cells can stably maintain pro-inflammatory or immunotolerant phenotypes (32). Signaling pathways involving ROS, TNF-α, IL-6 and lactate in the bone marrow microenvironment of osteoporotic (OP) bone may alter the accessibility of genes associated with the T-cell lineage by influencing the activity of epigenetic enzymes, thereby stabilizing the Th17 phenotype while making Tregs more prone to destabilization (33).

#### 6.2.2 Foxp3 stability is key to the durability of Tregs-mediated bone protection

Stable Tregs typically exhibit low levels of methylation at the FOXP3 promoter and conserved non-coding sequences, enabling sustained expression of Foxp3, CD25 and CTLA-4; whereas in an inflammatory environment, Tregs may undergo phenotypic drift characterized by decreased Foxp3 and increased expression of IL-17 or IFN-γ, transforming from protective cells into functionally unstable cells. Osteoporosis treatments that merely increase Tregs numbers without maintaining the epigenetic stability of Foxp3 may fail to achieve long-term bone-protective effects. Therefore, future Tregs-related research should simultaneously assess cell numbers, suppressive function and Foxp3 stability (57, 58).

#### 6.2.3 Non-coding RNAs may represent a crucial regulatory layer linking inflammatory signaling to T-cell differentiation

miR-155, miR-146a, miR-21, miR-326, miR-210 and others have been shown to participate in T-cell activation, Th17 differentiation, Tregs function or the negative feedback regulation of inflammation. Long non-coding RNAs (lncRNAs) and circular RNAs (circRNAs) may also regulate pathways such as STAT3, RORγt, Foxp3, NF-κB and PI3K/Akt via competitive endogenous RNA networks (138). Although research into non-coding RNAs in the OP-T cell axis remains insufficient to date, they may serve as important molecular biomarkers for explaining patient heterogeneity, differences across disease stages and variations in treatment response (139).

#### 6.2.4 Hypoxia and the bone marrow metabolic microenvironment can reshape T-cell effects

The bone marrow is not a homogeneous tissue; oxygen tension, nutrients and metabolic products vary across the endosteum, perivascular regions, fatty areas and regions adjacent to resorption pits. Hypoxia can activate HIF-1α, promoting glycolysis and favoring Th17 differentiation; conversely, fatty acid and ketone body metabolism may support the maintenance of Tregs or memory T cells (38). In age-related and diabetic osteoporosis, increased bone marrow fat, impaired vascular function and mitochondrial damage alter the metabolic substrates available to T cells, thereby influencing T-cell differentiation and cytokine production (40).

## 7 Mechanisms and translational implications of T-cell regulation in osteoporosis treatment

### 7.1 Cross-mechanisms between T-cell regulation and existing antiosteoporotic therapies

#### 7.1.1 Antiresorptive drugs and T-cell osteoimmunity

Bisphosphonates primarily induce osteoclast dysfunction or apoptosis by inhibiting the mevalonate pathway and blocking isopentenylation of small GTPases, their core targets are not T cells (121); however, nitrogen-containing bisphosphonates can influence the monocyte-osteoclast lineage and activate Vγ9Vδ2 T cells, indicating that some bone-targeting drugs may simultaneously alter the state of immune cells (122). Denosumab directly neutralizes RANKL and in theory, could simultaneously block RANKL signaling from osteoblasts, osteocytes and activated T cells; however, its primary clinical effect remains the inhibition of osteoclast formation (113, 140).

#### 7.1.2 Osteoanabolic drugs and T cells

Intermittent PTH administration promotes bone formation, but PTH may also participate in the regulation of bone metabolism via T cells and IL-17 (123). Under different dosing regimens, PTH may promote bone formation via osteoblasts and osteocytes, or, when maintained at persistently high levels, may enhance RANKL and bone resorption. Studies suggest that T-cell-derived Wnt10b may be involved in the osteogenic effects of PTH, whereas Th17/IL-17 may be involved in PTH-related bone resorption effects (124). Consequently, the immunological context of osteogenic drugs may influence therapeutic efficacy.

#### 7.1.3 Sclerostin antibodies and the immune microenvironment

Romosozumab enhances Wnt/β-catenin signaling by inhibiting sclerostin, thereby exerting both osteogenic and, to a certain extent, antiresorptive effects (125). Although its direct target is osteocyte-derived sclerostin, there is an overlap between Wnt signaling and immune cell function; T-cell-derived inflammatory factors may also influence osteoblasts’ response to Wnt signaling (126).Future studies could examine changes in Th17/Tregs cells, TNF-α and RANKL/OPG before and after romosozumab treatment to determine whether the immune microenvironment contributes to variations in its therapeutic efficacy.

#### 7.1.4 Vitamin D, calcium supplements and nutritional immunology

Vitamin D is not only involved in calcium and phosphorus metabolism but also exerts immunomodulatory effects, influencing dendritic cell maturation, Th17 differentiation and Tregs function. Vitamin D deficiency may exacerbate inflammatory responses and bone resorption, whereas adequate vitamin D status helps maintain bone mineralization and immune homeostasis (119). Proteins, omega-3 fatty acids, dietary fiber, antioxidant nutrients and trace elements may also influence bone health via the gut microbiota, short-chain fatty acids and T-cell metabolism (120).

### 7.2 Evaluation systems and study designs in clinical translation

#### 7.2.1 Establishing a T-cell bone immunophenotyping system

To effectively use T-cell subsets in the clinical translation of antiosteoporotic therapies, it is first necessary to establish a reproducible and interpretable immunophenotyping framework. Simply measuring the proportion of Th17 or Tregs cells in peripheral blood is insufficient to reflect the local state of the bone marrow (40); therefore, flow cytometry, serum cytokines, the RANKL/OPG ratio, bone turnover markers and imaging parameters should be integrated. Recommended baseline parameters include CD4+ T cells, CD8+ T cells, Th17, Tregs, Th1, Th2, γδ T-cell proportions and their activation markers (40, 128); functional markers should include levels of IL-17A, TNF-α, IFN-γ, IL-6, IL-10, TGF-β, RANKL and OPG; and bone metabolism markers should include CTX, TRAP5b, P1NP, BALP, OCN and 25(OH)D (42, 46, 63, 68, 141). These markers allow for a preliminary distinction between inflammatory resorption-dominant, Tregs-deficient, immunosenescence-associated and metabolic-inflammatory types of osteopenia.

#### 7.2.2 Emphasis on local bone marrow evidence

Whereas peripheral blood T-cell testing is convenient, the key pathological changes in osteoporosis occur in the bone marrow and the bone tissue microenvironment. Bone marrow T cells may differ significantly from peripheral blood T cells in terms of antigenic experience, tissue residence, metabolic status and cytokine release. Therefore, where ethically permissible, samples may be obtained during hip fracture surgery, joint replacement or bone marrow aspiration to analyze bone marrow T cell subsets, bone marrow plasma cytokines, and RANKL/OPG expression and spatial localization in bone tissue (128). If the bone marrow immune profile can be correlated with trabecular microstructure, cortical bone porosity and bone turnover markers, this will help to validate the causal relationship between T-cell abnormalities and bone loss.

#### 7.2.3 Designing a pathway for mechanistic validation

Many studies on T-cell involvement in osteopenia remain at the level of correlation; more rigorous causal validation is required in the future. Animal experiments may use T-cell-deficient mice, IL-17A-deficient mice, Foxp3-DTR mice, TCRδ-deficient mice, and T-cell-specific STAT3 or mTOR knockout models, combined with OVX, aging, high-glucose, glucocorticoid and immobilization models, to clarify the necessity and sufficiency of specific T-cell subsets in different types of OP (73, 80). *In vitro* experiments could establish co-culture systems involving T cells and osteoblasts, T cells and osteoclast precursors, and T cells and BMSCs, to distinguish between cell-contact-dependent effects and soluble factor effects.

#### 7.2.4 Focus on the time window and disease stage

The role of T cells in bone remodeling exhibits distinct stage-specific characteristics. In the early stages of bone injury, moderate inflammation is required to initiate repair; premature or excessive suppression of effector T cells may impair the clearance of necrotic tissue and the recruitment of repair cells. Conversely, in chronic osteoporotic states, sustained activation of Th17 cells, TNF+ T cells or senescent T cells leads to excessive bone resorption and inhibition of osteogenesis (51, 52). Therefore, therapeutic studies should set endpoints according to the disease stage: in the early stage, the focus should be on the resolution of inflammation and the initiation of osteogenesis; in the intermediate stage, on the coupling of resorption and osteogenesis; and in the long term, on bone mineral density, bone quality and fracture risk (63).

#### 7.2.5 Optimizing clinical trial endpoints

Antiosteoporotic studies targeting T cells should not rely solely on BMD as an endpoint. Improvements in BMD typically require a prolonged period, whereas immunomodulation may first manifest as a reduction in cytokines, improvements in bone turnover markers, alleviation of bone marrow inflammation and microstructural stabilization. It is recommended that clinical studies employ multi-tiered endpoints: primary endpoints could be BMD or fracture incidence; secondary endpoints could include P1NP, CTX, TRAP5b, RANKL/OPG, pain and quality of life scores; and mechanistic endpoints could include the Th17/Tregs ratio, T-cell activation markers, TCR clonal diversity, gut microbiota and short-chain fatty acids (128, 142). Only by integrating clinical endpoints with mechanistic endpoints can it be determined whether the intervention truly exerts a bone-protective effect via T-cell subsets.

#### 7.2.6 Constructing a multi-omics integrated model

The regulation of osteoporosis by T-cell subsets involves multiple levels, including transcriptional, epigenetic, proteomic, metabolic and microbiome aspects. Single-cell transcriptomics can elucidate the interactions between T-cell subsets and osteocyte subsets; spatial transcriptomics can reveal the spatial proximity of RANKL+ T cells, IL-17+ T cells and osteoclasts within bone tissue; metabolomics can reveal the effects of short-chain fatty acids, tryptophan metabolites, bile acids and lactate on T-cell fate; and proteomics can provide additional evidence at the cytokine and receptor levels (31, 55, 141, 143). In the future, machine learning could be used to establish predictive models of immune-bone metabolism, which could be employed to screen high-risk patients and predict treatment responses.

## 8 Research challenges and future directions

First, T-cell subsets do not constitute a fixed dichotomous structure, but rather a continuous, dynamic and tissue-specific functional spectrum. Traditional Th17/Tregs and Th1/Th2 classifications aid in understanding mechanisms, but cannot fully explain the differences in T-cell states observed in the bone marrow, gut, lymph nodes and peripheral blood (139). Future research should use single-cell RNA sequencing, single-cell ATAC sequencing, TCR sequencing and spatial transcriptomics to identify pathogenic T-cell subsets that are localized within the bone marrow microenvironment, the periosteum or the resorption interface, rather than relying solely on peripheral blood proportions to infer the intraosteoimmune status.

Second, osteoporosis (OP) is highly heterogeneous. The T-cell mechanisms underlying postmenopausal OP, age-related OP, diabetic OP, glucocorticoid-induced OP, disuse OP and inflammatory bone loss are not identical (89, 92, 112). A single patient may simultaneously exhibit estrogen deficiency, aging, gut microbiota dysbiosis, metabolic abnormalities and chronic inflammation. Future clinical research should perform immunophenotyping and develop integrated models combining the Th17/Tregs ratio, cytokine profiles, gut microbiota, bone turnover markers and imaging findings to determine which patients are more likely to benefit from T-cell-targeted therapies.

Third, treatment must balance between ‘suppressing inflammation’ and ‘preserving immune defense’. T cells are crucial for anti-infective and anti-tumor defense, as well as tissue repair; long-term systemic suppression of IL-17, TNF-α or JAK/STAT may carry risks such as infection, reduced tumor immune surveillance or weakened vaccine responses (144) OP treatment often continues for many years, necessitating higher safety standards than short-term anti-inflammatory therapy. Therefore, future efforts should focus on developing bone-targeted, local delivery, microenvironment-responsive and stage-specific regulatory technologies, rather than relying solely on systemic immunosuppression (145).

Fourth, there remains a gap between existing evidence from animal models and its translation to humans. OVX mouse or rat models can simulate estrogen deficiency, but cannot fully replicate the long-term chronic course of postmenopausal disease in humans; geriatric models, diabetic models and models with concomitant inflammation also have their respective limitations (94, 102). Human bone marrow samples are difficult to obtain, and peripheral blood T cells may not accurately reflect the status of intraosseous T cells. In future, a combination of bone marrow aspiration samples, bone tissue discarded during fracture surgery, organoids, bone-marrow-on-a-chip systems and humanized mouse models could be used to enhance the clinical relevance of mechanistic research.

Fifth, evaluation metrics should be expanded from bone mineral density alone to include bone quality and immune homeostasis. BMD measured by DXA remains a core clinical indicator; however, immunological interventions may primarily alter bone turnover, bone marrow inflammation and microstructure, rather than significantly increasing BMD in the short term. It is recommended that future studies simultaneously measure HR-pQCT, bone turnover markers, inflammatory cytokines, T-cell subsets, TCR clones, gut microbiota and metabolomics to comprehensively assess the true efficacy of T-cell regulation in treating OP (144, 145).

Overall, the future direction of T-cell subset-mediated regulation in OP treatment should shift from ‘identifying correlations’ to ‘validating causality’, from ‘blocking single cytokines’ to ‘patient stratification combined with local precision regulation’, and from ‘inhibiting bone resorption’ to ‘restoring osteoimmune homeostasis’. Only when the pathogenic subsets, therapeutic windows, delivery methods and safety thresholds have been clearly defined can T-cell-targeted strategies truly become an important adjunct to OP treatment.

## 9 Conclusions

T-cell subsets serve as a critical bridge linking immune inflammation to imbalances in bone remodeling. Th17 cells promote osteoclast formation via IL-17, RANKL, TNF-α and the NF-κB/MAPK/NFATc1 pathways, and they represent a key pathogenic subset in postmenopausal OP, inflammatory bone loss and certain metabolic bone diseases; Tregs cells inhibit osteoclastogenesis through Foxp3-, IL-10-, TGF-β-, CTLA-4-, and IDO-related mechanisms, thereby limiting inflammatory amplification and maintaining bone marrow immune homeostasis, and thus represent important protective subsets in the immune regulation against osteoporosis. Th1/Th2 cells, CD8+ T cells, γδ T cells, NKT cells and other unconventional T cells may also participate in the regulation of bone resorption and formation depending on the disease context and tissue microenvironment.

From a therapeutic perspective, simply inhibiting osteoclasts or promoting osteoblasts cannot fully account for the complex pathology of osteoporosis. Restoring the balance of T-cell subsets, reducing chronic low-grade inflammation, re-establishing Tregs-mediated immune tolerance, modulating the gut microbiota and improving the metabolic state of T cells may become important complementary approaches to traditional osteoporosis treatments. Future research should use single-cell omics, spatial transcriptomics, immunometabolomics and high-quality clinical cohorts to elucidate the T-cell pathogenic networks in different OP subtypes, and to develop precision immunological intervention strategies characterized by bone-targeting, stage-responsiveness and long-term safety.

## Glossary

AGEs: advanced glycation end products
Akt: protein kinase B
ALP: alkaline phosphatase
AMPK: AMP-activated protein kinase
BALP: bone-specific alkaline phosphatase
BMD: bone mineral density
BMP: bone morphogenetic protein
BMSCs: bone marrow mesenchymal stem cells
CD: cluster of differentiation
CD80/86: cluster of differentiation 80/86
circRNAs: circular RNAs
COL1A1: collagen type I alpha 1 chain
CTLA-4: cytotoxic T-lymphocyte-associated protein 4
CTLA-4-Ig: cytotoxic T-lymphocyte-associated protein 4 immunoglobulin
CTX: C-terminal telopeptide of type I collagen
CTSK: cathepsin K
DC-STAMP: dendritic cell-specific transmembrane protein
DXA: dual-energy X-ray absorptiometry
ERK: extracellular signal-regulated kinase
Foxp3: forkhead box P3
GATA-3: GATA-binding protein 3
GM-CSF: granulocyte-macrophage colony-stimulating factor
HIF-1α: hypoxia-inducible factor-1 alpha
HR-pQCT: high-resolution peripheral quantitative computed tomography
ICOS: inducible T-cell costimulator
IDO: indoleamine 2,3-dioxygenase
IFN-γ: interferon-gamma
IL: interleukin
IL-1β: interleukin-1 beta
IL-2: interleukin-2
IL-4: interleukin-4
IL-5: interleukin-5
IL-6: interleukin-6
IL-7: interleukin-7
IL-10: interleukin-10
IL-12: interleukin-12
IL-13: interleukin-13
IL-17: interleukin-17
IL-17A: interleukin-17A
IL-17F: interleukin-17F
IL-18: interleukin-18
IL-21: interleukin-21
IL-22: interleukin-22
IL-23: interleukin-23
JAK: Janus kinase
JNK: c-Jun N-terminal kinase
KLRG1: killer cell lectin-like receptor G1
lncRNAs: long non-coding RNAs
MAIT cells: mucosa-associated invariant T cells
MAPK: mitogen-activated protein kinase
MHC: major histocompatibility complex
miRNAs: microRNAs
MMP-9: matrix metalloproteinase-9
mTOR: mechanistic target of rapamycin
NFATc1: nuclear factor of activated T cells cytoplasmic 1
NF-κB: nuclear factor-kappa B
NKT cells: natural killer T cells
OCN: osteocalcin
OP: osteoporosis
OPG: osteoprotegerin
OVX: ovariectomy
P1NP: procollagen type I N-terminal propeptide
PD-1: programmed cell death protein 1
PGE2: prostaglandin E2
PI3K: phosphoinositide 3-kinase
PTH: parathyroid hormone
RAGE: receptor for advanced glycation end products
RANK: receptor activator of nuclear factor-kappa B
RANKL: receptor activator of nuclear factor-kappa B ligand
RORγt: retinoic acid receptor-related orphan receptor gamma t
ROS: reactive oxygen species
Runx2: runt-related transcription factor 2
SCFAs: short-chain fatty acids
STAT: signal transducer and activator of transcription
T-bet: T-box transcription factor 21
TCR: T-cell receptor
Tfh cells: T follicular helper cells
TGF-β: transforming growth factor-beta
Th cells: T helper cells
Th1: T helper 1
Th2: T helper 2
Th9: T helper 9
Th17: T helper 17
Th22: T helper 22
TNF-α: tumor necrosis factor-alpha
TRAF6: tumor necrosis factor receptor-associated factor 6
TRAP: tartrate-resistant acid phosphatase
TRAP5b: tartrate-resistant acid phosphatase 5b
Tregs: regulatory T cells
Wnt: Wingless-related integration site.
